# Role of Mannose-Binding Lectin Deficiency in HIV-1 and *Schistosoma* Infections in a Rural Adult Population in Zimbabwe

**DOI:** 10.1371/journal.pone.0122659

**Published:** 2015-04-01

**Authors:** Rutendo B. L. Zinyama-Gutsire, Charles Chasela, Hans O. Madsen, Simbarashe Rusakaniko, Per Kallestrup, Michael Christiansen, Exnevia Gomo, Henrik Ullum, Christian Erikstrup, Shungu Munyati, Edith N. Kurewa, Babill Stray-Pedersen, Peter Garred, Takafira Mduluza

**Affiliations:** 1 Faculty of Health Sciences, School of Public Health, University of the Witwatersrand, Johannesburg, South Africa; 2 Medical Research Council of Zimbabwe, Ministry of Health and Child Welfare, Harare, Zimbabwe; 3 Letten Research Foundation, Harare, Zimbabwe; 4 Tissue typing Laboratory, Department of Clinical Immunology, Rigshospitalet, Copenhagen, Denmark; 5 University of Zimbabwe, College of Health Sciences, Harare, Zimbabwe; 6 Centre for Global Health, Department of Public Health, Aarhus University, Aarhus, Denmark; 7 Department of Clinical Biochemistry, Immunology and Genetics, Statens Serum Institute, Copenhagen, Denmark; 8 Department of Medical Laboratory Sciences, College of Health Sciences, University of Zimbabwe, Harare, Zimbabwe; 9 Laboratory of Molecular Medicine, Department of Clinical Immunology, Copenhagen University, Rigshospitalet, Copenhagen, Denmark; 10 Biomedical Research and Training Institute, Harare, Zimbabwe; 11 Division of Women and Children, Rigshospitalet Oslo University Hospital and University of Oslo, Oslo, Norway, and Letten Research Foundation, Harare, Zimbabwe; 12 University of Zimbabwe, Biochemistry Department, Harare, Zimbabwe, and Department of Laboratory Medicine and Medical Sciences, University of KwaZulu Natal, Durban, South Africa; University of Leicester, UNITED KINGDOM

## Abstract

**Background:**

Polymorphism in the *MBL2* gene lead to MBL deficiency, which has been shown to increase susceptibility to various bacterial, viral and parasitic infections. We assessed role of MBL deficiency in HIV-1 and schistosoma infections in Zimbabwean adults enrolled in the Mupfure Schistosomiasis and HIV Cohort (MUSH Cohort).

**Methods:**

HIV-1, *S*. *haematobium* and *S*. *mansoni* infections were determined at baseline. Plasma MBL concentration was measured by ELISA and *MBL2* genotypes determined by PCR. We calculated and compared the proportions of plasma MBL deficiency, *MBL2* structural variant alleles *B* (codon 54A>G), *C* (codon 57A>G), and *D* (codon 52T>C) as well as *MBL2* promoter variants *-550(H/L)*, *-221*(*X/Y)* and *+4*(*P/Q)* between HIV-1 and schistosoma co-infection and control groups using Chi Square test.

**Results:**

We assessed 379 adults, 80% females, median age (IQR) 30 (17–41) years. HIV-1, *S*. *haematobium* and *S*. *mansoni* prevalence were 26%, 43% and 18% respectively in the MUSH baseline survey. Median (IQR) plasma MBL concentration was 800μg/L (192-1936μg/L). Prevalence of plasma MBL deficiency was 18% with high frequency of the *C* (codon 57G>A) mutant allele (20%). There was no significant difference in median plasma MBL levels between HIV negative (912μg/L) and HIV positive (688μg/L), p = 0.066. However plasma MBL levels at the assay detection limit of 20μg/L were more frequent among the HIV-1 infected (p = 0.007). *S*. *haematobium* and *S*. *mansoni* infected participants had significantly higher MBL levels than uninfected. All *MBL2* variants were not associated with HIV-1 infection but promoter variants *LY* and *LL* were significantly associated with *S*. *haematobium* infection.

**Conclusion:**

Our data indicate high prevalence of MBL deficiency, no evidence of association between MBL deficiency and HIV-1 infection. However, lower plasma MBL levels were protective against both *S*. *haematobium* and *S*. *mansoni* infections and *MBL2* promoter and variants *LY* and *LL* increased susceptibility to *S*. *haematobium* infection.

## Introduction

HIV-1 and schistosomiasis co-infections are very common in Africa and have been reported in several studies [1–6]. Sub-Saharan Africa is the region hardest hit by the HIV/AIDS pandemic where 63% of the 33 million infected people live [7]. HIV infection has remained a major public health challenge since its discovery in 1983 [8]. The HIV pandemic is still ravaging most parts of Southern African countries with current prevalence in these countries between 10–20% [7]. Several reports indicate that individuals worldwide differ in their susceptibility to HIV infection and it is widely agreed that genetic polymorphisms in the host genes important in immune regulation can increase or reduce the risk of HIV infections [9, 10]. An understanding of the immunological factors fuelling the HIV-1 epidemic in Africa is very important in an effort to curb the HIV-1 scourge.

Schistosomiasis is one of the neglected tropical diseases, which the World Health Organization is targeting for elimination [11–13]. An estimated 85% of the world’s estimated 200 million people with schistosomiasis live in Sub-Saharan Africa [11, 13]. *S*. *haematobium* is associated with urogenital schistosomiasis characterised by severe pathological conditions including hematuria and bladder cancer and *S*. *mansoni* causes intestinal schistosomiasis characterised by chronic or intermittent abdominal pain bleeding from gastro-oesophageal varices and bloody stool [14]. Schistosomes are complex multi-cellular helminths with several developmental stages well documented in the human host [14].

Mannose-Binding Lectin (MBL) is a key component of the innate immune system and polymorphism in the *MBL2* gene and promoter region lead to MBL deficiency [15–17]. MBL deficiency is associated with impaired function of the innate immune system and leads to increased susceptibility to several infections [18–20]. MBL acts as an opsonin by binding to sugar groups naturally found on the surface of various infectious bacteria, viruses and parasites and activates the complement system [21, 22] through associating with MBL-associated serine proteases MASP-1, MASP-2 and MASP-3 [23–25]. Sub-Saharan Africa has a high burden of viral [26–28], bacterial [27–29] and parasitic infections [12, 13, 27]. Assessment of polymorphisms in the *MBL2* gene and promoter region to determine functional MBL deficiency has been carried out in different populations with few studies in Sub-Saharan Africa [17, 30, 31]. Schistosomes carry sugar molecules or glycoconjugates on the surface of all their developmental stages [32, 33] and these glycoconjugates interact with innate immune recognition molecules including MBL [34–36]. *In vitro* studies have demonstrated compliment mediated killing of all stages of the schistosome parasite [35].

Several studies have been conducted worldwide [37–43] including several African populations [17, 30, 44–48] looking at *MBL2* genetic variants and plasma/serum MBL concentration and their association to increased infection susceptibility. The role of MBL deficiency in infections has been shown to be different across different infections [44]. For example MBL deficiency has been shown to increase risk of recurrent respiratory infections [49] and malaria [50] whilst being protective in TB [44], leprosy [51] and leishmaniasis [52, 53]. Results on the role of MBL deficiency in HIV infections are conflicting [54, 55]. In addition, studies conducted in Sub-Saharan Africa, in Mozambique [17, 56, 57], South Africa [48, 58] and Zimbabwe [31] have been done in children and none among adults. One study reports investigations on MBL deficiency and schistosoma infections in Nigeria [59].

In this paper we describe the role of plasma MBL deficiency and *MBL2* polymorphism in HIV-1, *S*. *mansoni* and *S*. *haematobium* infections in Zimbabwean adults enrolled in the MUSH cohort. The infection rates in Zimbabwe are currently 15% for HIV-1 [60] and 40% and 20% for S*chistosoma haematobium* and *Schistosoma mansoni* respectively [61]. *S*. *haematobium* is the most common species followed by *S*. *mansoni* in Zimbabwe [61–64]. In the context of public health, it is important to understand the role of MBL deficiency in these infections in our adult population.

## Materials and Methods

### Study design and study population

**Fig 1:**
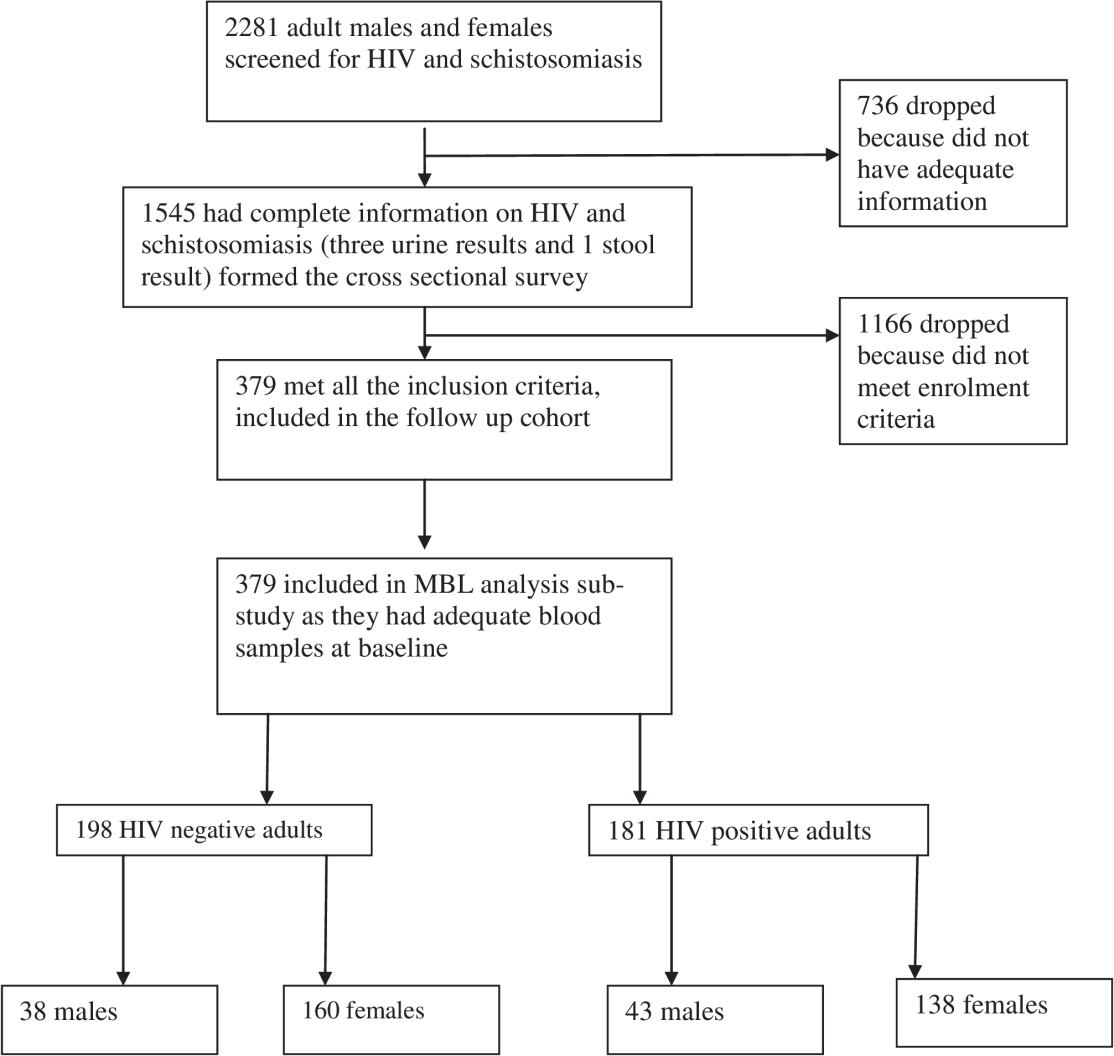
Participant Flow diagram. Flow chart showing the recruitment procedures for the adult cohort. A total of 2281 community dwelling rural adult males and females were screened for HIV and schistosomiasis, 379 males and females who met the inclusion criteria were enrolled and their baseline blood samples used for MBL plasma and *MBL2* genotype analysis.

This was a sub-study of the Mupfure Schistosomiasis and HIV Cohort (MUSH Cohort) established in 2001 aimed at studying the immunological interactions between HIV-1 and schistosomiasis infections [35, 36, 38, 44, 45]. We used plasma and whole blood samples collected at baseline in 2001–2002 to determine prevalence of MBL deficiency in this population. Details of the MUSH Cohort and procedures are described elsewhere [1, 2, 65]. Briefly, recruitment into the MUSH study, sample collection and laboratory assays took place between October 2001 and November 2007. A total of 1574 adult males and females were screened for HIV and schistosomiasis infections at baseline (Fig. 1) and 379 met the MUSH eligibility criteria: adult males/females above 18 years old, HIV positive or negative, positive for *S*. *haematobium* or *S*. *mansoni* or both or uninfected, no active TB infection, non-pregnant women and not currently on schistosomiasis treatment. After screening for HIV-1 and schistosomiasis status, participants were categorised into four groups: HIV-1 only, schistosomiasis only, co-infected with both diseases and controls with none of these two infections.

This sub-study was approved by the National Research Ethics Committee of the Medical Research Council of Zimbabwe (MRCZ/A/1770) and the University of Witwatersrand Human Research Ethics Committee (M130348). Permission was given by the Provincial Medical Director of Mashonaland Central Province and the District Medical Officer of Shamva District, the village headmen and at village meetings. All individuals in the main MUSH study gave written informed consent for specimen collection, storage and future laboratory studies.

### Laboratory procedures

#### HIV testing

Participants were screened for *S*. *haematobium*, *S*. *mansoni* and HIV-1 as described earlier (1, 65). Briefly, HIV pre- and post-test counselling was done in the participants’ native language (Shona) by qualified medical personnel. A rapid HIV-1/2 test kit was used on a dry blood spot in the field (Determine, Abbott Laboratories, Tokyo, Japan), followed by two different rapid HIV tests Oraquick (by Orasure) and Serodia (by Fujirebio) for all who tested initially positive. No discrepancies in results were found between the initial Determine HIV test and the two subsequent ELISAs. Strict confidentiality was maintained throughout the study. All those who requested their HIV results were given after post-test counselling from the nursing staff.

#### Schistosoma parasitology

Microscopic examination of fixed-volume urine samples filtered on Nytrel filters (VesterGaard Frandsen) was used to identify and quantify eggs of *S*. *haematobium* by the syringe urine filtration technique [66]. Due to the diurnal and day-to-day variation in egg output, the urine samples were collected on 3 consecutive days [67, 68]. The modified formol-ether concentration technique was used on 1 stool sample from each participant to detect eggs of *S*. *mansoni* and other helminth or parasites [69]. Levels of Circulating Anodic Antigen (CAA) were measured in serum samples using an ELISA assay, to complement parasitological detection methods. CAA originates from the parasite gut and is used as a marker of an active schistosome infection [70].

#### Determination of plasma MBL concentration

Baseline plasma samples stored at -20°C were used for measurement of plasma MBL concentrations using the double enzyme immuno-assay (EIA) (Antibody Shop, Denmark) as described previously [30, 71]. Micro titre plates (Maxisorp, NUNC, Denmark) were coated with a mouse anti-human MBL monoclonal antibody, clone 131–1, IgG1, Kappa (Antibody Shop, Denmark). Plasma samples were diluted 1/25 and 1/400 in Tris/HCL buffer (Bie and Bernsten) containing EDTA (Bie and Bernsten) and 0,05% Tween 20 (Sigma) [71]. A pool of EDTA plasma with a known concentration of MBL was used as the standard. Biotinylated clone 131–1 anti-MBL (Antibody Shop, Denmark) was used as the detection antibody. Horseradish peroxidase labelled streptavidin (Amersham, UK) was added. The substrate uses O-phenylene diamine (OPD) (DakoCytomation) hydrochloride in citrate-phosphate (Bie and Bernsten) buffer pH 5.0, the colour reaction was stopped by addition of sulphuric acid (Sigma). Parallel control plates were coated with equivalent amounts of mouse IgG1 and processed as above. Parallel plates are done in order to reveal binding of rheumatoid factors, anti mouse immunoglobulins and non-specific binding of MBL interfering in the system. Optical density (OD) was read on an ELISA reader (MR5000/7000, Dynatech Laboratories, Denmark) at 490 nm with reference filter 630nm. Final MBL values are given as μg MBL per litre. To determine prevalence of MBL deficiency, plasma MBL concentration was categorised into normal (above 500μg/L), intermediate (100μg/L- 500μg/L) and deficient (below 100μg/L) [30, 72–74].

#### MBL genotyping

Genomic deoxyribonucleic acid (DNA) was used for *MBL2* genotyping. The DNA was extracted from frozen baseline Peripheral Blood Mononuclear Cells (PBMCs) using the standard salting out procedure [75]. The *MBL2* genotypes and promoter region alleles were detected by allele-specific oligonucleotide PCR (ASO-PCR) where specific sequences were used for each allele [42]. Briefly, the PCR master mix was made by mixing the following: 400μl sterile water (H/S Apoteket, Rigshospital, Copenhagen), 150μl of 1.5mM MgCl2 (Invitrogen, Denmark), 150μl at 0.07mM final concentration of each deoxynucleotide triphosphate (dNTPs) (Amersham Biosciences, UK Limited), 500 μl PCR Buffer containing 50mM KCL, 10 mM Tris-HCl (pH 8.3), 0.001% (w/v) gelatin p.H.8.3 (Invitrogen, Denmark), 250μl glycerol at 5% final concentration, 50μl cresol red at final concentration 100μg/μl. Cresol red (Invitrogen, Denmark) was added as a colour marker. The PCR Master mix could be kept at 4°C for 2 weeks.

Then a total of 12 primer solutions (DNA Technology) were prepared for each sample (S1 Table). The set of 12 primers for *MBL2* variants and promoter region mutations were prepared by mixing 5´ sense and 3´ sense primers with water according to the manufacturer’s instructions. Final concentration of each primer was 0.25uM for each specific primer. Primers for 5 and 3 sense exon 4 of the *MBL2* gene (DNA Technology, Denmark) were included in each PCR reaction as an internal positive control. PCR primers were pre-aliquoted in 5μl volumes into the PCR plates and the plates could be kept at 4°C for 1 week.

MBL PCR and sample reaction mixtures were then prepared as 10μl volumes containing 30ng genomic DNA and 0.25μmM of the specific primer, 54 μl of PCR mastermix, 3,5μl sample DNA (if the DNA concentration was 0,3μg/μl), 31μl sterile water, and 0.25 Units of Platinum *Taq* DNA polymerase (Invitrogen, Denmark) in each 90μl initial reaction mixture volume. PCR reaction mixture was mixed and added to the 12 wells, clearly marked for each sample, which contained 5μl of different sets of primers for the genotyping and promoter typing. Only eight samples could be run on each PCR plate. Plates were put in a plate micro centrifuge at 3000rpm for 1 minute to spin down the well contents. Plates were tightly sealed with foil paper.

DNA was then amplified by PCR in a programmed thermocycler (Gene Amp PCR Systems 9600, Perkin Elmer). All PCRs were initiated by a 2-min denaturation step at 94°C and completed by a 5-min extension step at 72°C. The temperature cycles for the PCR were as follows: 10 cycles of 10 s at 94°C and 60 s at 65°C, and a further 20 cycles of 10 s at 94°C, 50s at 61°C, 30s at 72°C and 2 minutes at 25°C. Gel electrophoresis was done on the PCR products on the same day or kept at 4°C for a maximum 1 week. The PCR products were separated by a 2% agarose gel (Invitrogen) electrophoresis run in an electrophoresis chamber (Sub-cell Biorad) at 150V, 400mA for 32 minutes. The gels were then carefully transferred to the UV Transilluminator (Syngene Synoptics Ltd, Denmark) where a photograph of the gel was taken and used to determine the *MBL2* genotype of each sample. *MBL2* genotypes *A/O*, *O/O* and *XAYO/YOYO* were classified as MBL deficient producers and the normal *MBL2* denoted *A/A*. *MBL2* single nucleotide polymorphisms (SNPs) in the form of structural variants named *B* (codon 54, rs1800450 A>G), *C* (codon 57, rs1800451 A>G) and *D* (codon 52, rs5030737 T>C), as well as the regulatory promoter region variants *H/L* (-550, rs11003125 G>C), *X/Y* (-221, rs7096206 C>G) and *PQ* (+4, rs7095891 T>C) [76, 77] were determined [42]. All the single nucleotide polymorphisms (SNPs) and their reference sequence numbers are found on the online database http://www.ncbi.nlm.nih.gov/snp/.

### Statistical analysis

All statistical analysis were done using Stata 11 statistical package (STATA Corp, Timberlake Consultants). Normality of plasma MBL levels was checked graphically. Non-parametric tests (Mann-Whitney and Kruskal-Wallis) were used for comparison of median plasma MBL concentrations by age, gender, HIV-1 and schistosoma infection status. Differences in proportions of MBL deficiency between males and females and infection status were done using Chi Square or Fisher’s exact tests and confidence intervals were calculated using binomial exact methods. The frequencies of the *MBL2* alleles were obtained by direct gene counting. Conformation to the Hardy-Weinberg Equilibrium was determined using the Chi Square test in the SHEsis online programme [78, 79]. For *MBL2* genotype analysis, normal homozygous *MBL2* was denoted as *A/A*. The variant genotypes *A/B* (A>G), *A/C* (G>A), *A/D* (C>T) were grouped together as *A/O* heterozygous *MBL2* as they all give low plasma MBL levels and all homozygous and compound homozygotes were grouped together as *O/O*. The differences in frequency of the *MBL2* genotypes, promoter region variants and haplotypes between infection status groups were determined using the Chi Square test or Fisher’s exact tests.

## Results

A total of 379 plasma and whole blood samples were available for MBL analysis. Median age (IQR) of the participants was 30 (17 to 41) years old and the majority were females (80%). Baseline demographic characteristics of this study population and prevalence of HIV-1, *S*. *haematobium* and *S*. *mansoni* among the 1545 participants screened during the main MUSH study have been described elsewhere [65].

### Prevalence of HIV-1, *S*. *haematobium* and *S*. *mansoni* in the MBL sub-study

The prevalence of HIV-1, *S*. *haematobium*, *S*. *mansoni* and co-infection with both schistosma species among the 379 participants used for the MBL sub-study was 52%, 58%, 7% and 10%, respectively (Table 1).

**Table 1:** Comparison of median plasma MBL concentrations between groups.

**Variable**	**n**	**Median MBL levels** μ**g/L**	IQR μg/L	p-value
**Sex**				
Males	76	824	159–1968	
Females	302	800	200–1936	0.561
**Age groups**				
<25 years old	82	920	209–2608	
>25 years old	297	776	166–1864	0.202
**HIV status**				
Positive	197	688	147–1904	
Negative	181	912	272–1968	0.065
**Schistosomiasis infection status**				
No infection	89	656	139–1152	
*S*. *haematobium* only	205	912	213–2320	
*S*. *mansoni* only	24	1016	258–2192	
Coinfected with both species	37	688	297–2432	0.036
**Schistosomiasis and HIV status**				
*S*. *haematobium*+/ HIV+	102	824	151–2160	
*S*. *haematobium*+/ HIV-	103	944	251–2496	
*S*. *haematobium*-/ HIV+	44	656	65–1176	
*S*. *haematobium*-/ HIV-	45	707	163–1152	0.037
***S*. *haematobium* infection only**				
*S*. *haematobium positive*	205	912	213–2320	
*S*. *haematobium negative*	89	656	139–1152	0.006
**Circulating anodic antigen**				
Above 40pg/ml (CAA positive)	293	848	161–2232	
Below 40pg/ml (CAA negative)	63	784	346–1152	0.308
**Eggs per 10ml urine, *S*. *Haematobium* infection**				
0 (no infection)	108	705	145–1264	
<10 (light infection)	123	896	230–2112	
10-<50 (medium infection)	55	1408	182–2528	
>50 (heavy infection)	9	295	87–2816	0.109

The concentration of MBL in plasma was measured in μg/L. Differences in median plasma MBL concentration in the different infection status categories were analysed by using non-parametric tests Mann-Whitney for two categories and Kruskal-Wallis for three or more categories.

### Plasma MBL levels

Results for plasma MBL concentration are available for 378 individuals; one sample repeatedly gave inconsistent plasma level results and was dropped from this analysis. The median (IQR) plasma MBL concentration in all the investigated individuals was 800μg/L (192–1936μg/L). There was no difference in the median plasma MBL concentration between males and females (p = 0.553) and by to age (p = 0.204) (Table 1).

### Association between HIV-1, schistosoma infections and plasma MBL concentration

There was no difference in the median plasma MBL concentration between HIV infected and uninfected individuals (p = 0.065). However, there were 37 participants with plasma MBL levels at the assay detection limit of 20μg/L. This level of plasma MBL was found more frequently among HIV-1 infected 14% (27 of 196, 95% CI 8.4–18.2), compared with HIV-1 uninfected 6% (10 of 181, 95% CI 2.7–9.9) (χ^2^ = 7.24, p = 0.007), indicating a possible role of MBL deficiency in HIV-1 infection.

There was a significant difference (p = 0.037) in median plasma MBL levels between the four schistosoma infection status groups, namely, no infection, *S*. *haematobium* only, *S*. *mansoni* only and those co-infected with both species. Those with *S*. *mansoni* infection only had the highest median plasma MBL level of 1016μg/L followed by those with *S*. *haematobium* only median MBL level 912μg/L, p = 0.037 (Table 1). There were also significant differences in median plasma MBL levels between the four HIV-1 and *S*. *haematobium* co-infection groups, with those positive for *S*. *haematobium* and negative for HIV having the highest median plasma MBL level (944μg/L, p = 0.037, Table 1). Further comparison was done between the *S*. *haematobium* infected and uninfected individuals after excluding those with *S*. *mansoni* or co-infected with both species. There was a significant difference in plasma MBL levels, those *S*. *haematobium* infected having higher median MBL levels than the uninfected, p = 0.006 (Table 1) indicating protective role low plasma MBL levels. There was no difference in plasma MBL levels when comparison was done between CAA levels groups above or below 40pg/ml (p = 0.381) and four egg count groups among those with *S*. *haematobium* infection only (p = 0.114, Table 1).

### Association between HIV-1, schistosoma infections and plasma MBL deficiency

Plasma MBL concentration was categorised into normal (above 500μg/L), intermediate (100μg/L- 500μg/L) and deficient (below 100μg/L) (Table 2) (72, 73). The prevalence of plasma MBL deficiency in all participants analysed was 18% (67 of 378, 95% CI: 14–22%), 20% (77 of 378, 95% CI: 16–24%) had intermediate plasma MBL levels and 62% (234 of 378, 95% CI: 56–66%) had normal plasma MBL levels above 500μg/L. There was no difference in proportion of males with MBL deficiency 20% (15 of 76, 95% CI: 11–30%) and females with MBL deficiency 17% (52 of 302, 95% CI: 13–22%).

**Table 2:** Distribution of participants between three plasma MBL levels, participants stratified according HIV, *S*. *haematobium* and *S*. *mansoni* infection and co-infection status.

HIV status	n	Normal MBL levels	Reduced MBL levels	Deficient MBL levels	P value
HIV positive	197	113 (57%)	41 (21%)	43 (22%)	
HIV negative	181	121 (67%)	36 (20%)	24 (13%)	0.070
***S*. *haematobium* infection**					
*S*. *haematobium* positive	205	131 (64%)	38(19%)	36 (17%)	
*S*. *haematobium* negative	89	51 (57%)	17(19%)	21 (24%)	0.446
**Schistosoma co-infection**					
No infection (controls)	89	51(57%)	17 (195)	21(24%)	
*S*. *haematobium* only	205	131(64%)	38(19%)	36(18%0	
*S*. *mansoni* only	24	15(63%)	6(25%)	3(13%)	
Co-infected with both species	37	23(62%)	11(30%)	3(8%)	0.351
**HIV and** ***S*. *haematobium* co-infection**					
HIV+/*S*. *haematobium*+	102	64(63%)	17(17%)	21(21%)	
HIV+/*S*. *haematobium*-	44	23(52%)	8(18%)	13(30%)	
HIV-/*S*. *haematobium*+	103	67(65%)	21(20%)	15(15%)	
HIV-/*S*. *haematobium*-	45	28(62%)	9(20%)	8(18%)	0.546

Prevalence of MBL deficiency, plasma MBL concentration was categorised into normal (above 500μg/L), intermediate (100μg/L- 500μg/L) and deficient (below 100μg/L), analysed by the Chi Square or Fisher’s exact tests, n = 378.

We found no difference in distribution frequency of participants according to HIV (p = 0.070) and among *S*. *haematobium* infected and uninfected participants after excluding *S*. *mansoni* and co-infections (p = 0.446) between the three plasma MBL levels categories, normal, reduced and deficient (Table 2). There was also no difference when a similar analysis was done among the schistosoma infection status groups (p = 0.351) and among HIV and *S*. *haematobium* co-infection groups (p = 0.546).

### *MBL2* polymorphism

*MBL2* genotyping was successfully done on 366 samples out of 379 and these were used in this analysis, the other samples could not be amplified. All the known *MBL2* coding alleles, wild-type *A/A*, *B* (codon 54, rs1800450 A>G), *C* (codon 57, rs1800451 A>G) and *D* (codon 52, rs5030737 T>C), were found in this study population. In the *MBL2* coding region, 233/366 (64%) were classified as *A/A* genotype, 3 (0.8%) were *A/B*, 117 (32%) were *A/C*, 1 (0.3%) *A/D* and 12 (3%) *C/C* (Table 3). There were neither *B/B*, *C/D*, *D/D* nor *B/D* variants. The frequency of *MBL2* genetic variants was 36% and 64% had the normal homozygous *A/A* genotype. The normal homozygous *MBL2*, *A/A* genotype was found at frequencies of 64% (233 of 366, 95% CI:58.5–68.5%), heterozygous MBL2 *A/O* variants at 33% (121 of 366, 95% CI: 28.3–38.1) and homozygous *O/O* variants at 3.3% (12 of 366, 95% CI:1.7–5.7%) (Table 3). All the *MBL2* SNPs detected among the HIV negative in this study were in Hardy-Weiburg Equillibrium (structural alleles p = 0.400, *-550H/L* p = 0.820, *-221Y/X* p = 0.550 and *+4P/Q* p = 0.170 (S2 Table).

**Table 3:** Gene, promoter alleles and haplotype frequencies obtained for *MBL2* polymorphism among the enrolled Zimbabwean participants.

	N	Frequency%
**Exon 1 allele**		
*A* normal	587	80
*B(A>G)*	3	0.4
*C(G>A)*	141	20
*D(C>T)*	1	0.1
***Promoter alleles***		
*-550H*	38	5
*-550L*	694	95
*-221X*	134	18
*-221Y*	598	82
*+4P*	343	47
*+4Q*	389	53
***MBL2* Exon 1 genotype**		
*AA* wild-type	233	64
*AB (A>G)*	3	0.8
*AC(G>A)*	117	32
*AD (C>T)*	1	0.2
CC *(C>T)*	12	3
**Promoter region genotypes**		
*-550HH*	1	0.3
*-550HL*	37	10.1
*-550LL*	328	89.6
*-221YY*	242	66.1
*-221XY*	112	30.0
*-221XX*	12	3.2
*+4PP*	74	20.2
*+4QQ*	97	26.5
*+4PQ*	195	53.3
***MBL2* Haplotypes**		
1. MBL2*LYPA	260	35.5
2. MBL2*LYQA	183	25.0
3. MBL2*LYQC	108	14.6
4. MBL2*LXPA	58	7.9
5. MBL2*LXQA	45	6.1
6. MBL2*HYPA	41	5.6
7. MBL2*LXQC	31	4.2
8. MBL2*LXPC	2	0.3
9. MBL2*LYQB	2	0.3
10. MBL2*HYPD	1	0.1
11. MBL2*LYPB	1	0.1

*MBL2* gene and allele frequencies obtained by direct gene counting, frequencies expressed as percentages.

### Frequency of *MBL2* genotypes and haplotypes

*MBL2* wild-type genotype *A/A* had the highest frequency of 64% (233 of 366, 95% CI: 77.1–83%) and genotype *A/D* the lowest frequency of 0.1% (1of 366, 95% CI 0.003–0.76, Table 3). All the currently known *MBL2* promoter region alleles, *-550H*, *-550L*, *-221Y*, *-221X*, *+4P* and *+4Q*, were detected. Promoter region allele *L* had the highest frequency 95% (694 of 732, 95% CI: 92.9–96.3%) compared to 5% (38 of 732, 95% CI: 3.69–7.1%) for the *H* allele (Table 3). The *LL MBL2* promoter region genotype (89%) had the highest frequency and *HH* (0.3%) the lowest frequency. Eleven different haplotypes were detected in these individuals including three rare haplotypes namely *LXQA*, *LXQC* and *LXPC*. Haplotype *LYPA* had the highest frequency (35%), followed by *LYQA* and *LYPB*, *LYPC* and *HYPD* the lowest (0.1%) (Table 3). MBL2 genotypes *A/A* showed the highest levels of plasma MBL concentrations and *O/O* genotypes had the lowest (S3 Table).

### Association between HIV-1, schistosoma infections and *MBL2* genotypes and promoter region genotypes

Analysis was done to determine if *MBL2* genetic and promoter region variants were associated with HIV-1 infection. The distribution of the three genotypes (*A/A*, *A/O* and *O/O*) did not differ between the HIV-1 infected and uninfected (p = 0.429, Table 4). The *MBL2* haplotypes were further combined and subdivided into three groups namely genotypes that give normal plasma MBL levels (*YA/YA*, *YA/XA*), intermediate levels (*XA/XA*, *YA/YO*) and deficient levels (*XA/YO*, *YO/YO*) (20, 77). There was no difference in distribution frequency when comparison was done among the HIV-1 strata and the three *MBL2* genotype groups *YAYA/YAXA*, *XAXA/YAYO* and *XAYO/YOYO* (p = 0.347, Table 4). None of these *MBL2* genotypes and promoter variants were associated with HIV-1 infection (Table 4).

**Table 4:** Distribution of participants between *MBL2* genotypes and promoter region haplotypes, participants stratified according to HIV-1 infection status.

***MBL2* genotypes/ haplotypes**	n	HIV negative	HIV positive	P value
*A/A*	233	117 (50%)	116 (50%)	
*A/O*	121	55 (45%)	66 (55%)	
*O/O*	12	4 (33%)	8 (67%)	0.429
*YAYA/YAXA*	223	113(51%)	110(49%)	
*XAXA/YAYO*	131	59(45%)	72(55%)	
*XAYO/YOYO*	12	4(33%)	8(67%)	0.347
**Promoter genotypes**				
*-550HH*	1	1(100%)	0(0%)	
*-550HL*	37	22(59%)	15(41%)	
*-550LL*	328	153(47%)	175(53%)	0.141
*-550HY*	31	19(61%)	12(39%)	
*-550LY*	326	154(47%)	172(53%)	
*-221LX*	9	3(33%)	6(67%)	0.229
*-221YY*	242	120(50%)	122(50%)	
*-221XY*	112	52(46%)	60(54%)	
*-221XX*	12	4(33%)	8(67%)	0.521
*-550HY*	38	23(61%)	15(39%)	
-221LY*LX	328	153(47%)	175(53%)	0.105
*+4C/C(PP)*	74	37(50%)	37(50%)	
*+4TT(QQ)*	97	55(57%)	42(43%)	
*+4C/TPQ*	195	98(50%)	97(49%)	0.545

This analysis was done using the Chi Square and Fisher’s exact tests, n = 366

We found no association between *MBL2* genotypes and PQ promoter genotypes and schistosoma infections (Table 5). However, there was a significant association between *S*. *haematobium* and *S*. *mansoni* infections and *LL* (p = 0.051) and LY*LX (p = 0.026) promoter genotypes. Therefore promoter region genotypes *LL*, *LY* and *LX* which encode for low plasma MBL levels were significantly associated with *S*. *haematobium* and *S*. *mansoni* infections.

**Table 5:** Distribution of participants between *MBL2* genotypes and promoter region haplotypes according to schistosoma infection status.

***MBL2* Genotype/ Haplotype**	N	No infection (controls)	***S*. *haematobium*** only	***S*. *mansoni*** only	Coinfected with both species	P value
*A/A*	216	51(24%)	128(59%)	15(7%)	22(10%)	
*A/O*	117	33(28%)	62(53%)	9(8%)	13(11%)	
*O/O*	11	4(36%)	6(55%)	0(0%)	1(9%)	0.893
*YAYA/YAXA*	208	49(23%)	123(59%)	14(7%)	22(11%)	
*XAXA/YAYO*	125	35(28%)	67(54%)	10(8%)	13(10%)	
*XAYO/YOYO*.	11	4(36%)	6(55%)	0(0%)	1(9%)	0.903
**Promoter region genotypes**						
*-550HH*	1	0(0%)	1(100%)	0(0%)	0(0%)	
*-550HL*	34	15(44%)	18(53%)	0(0%)	1(3%)	
*-550LL*	309	73(24%)	177(57%)	24(8%)	35(11%)	0.051
*-550HY*	28	14(50%)	13(46%)	0(0%)	1(4%)	
*-221LY*	307	72(24%)	178(58%)	23(7%)	34(11%)	
*-221LX*	9	2(22%)	5(56%)	1(11%)	1(11%)	0.072
*-221YY*	228	65(29%)	122(54%)	14(6%)	27(12%)	
*-221XY*	106	21(20%)	68(64%)	9(8%)	8(8%)	
*-221XX*	10	2(20%)	6(60%)	1(10%)	1(10%)	0.351
*-550HY*	35	15(43%)	19(54%)	0(0%)	1(3%)	
-221LY*LX	309	73(24%)	177(57%)	24(8%)	35(11%)	0.026
*+4PP*	69	16 (23%)	43(62%)	3(4%)	7(10%)	
*+4QQ*	88	17(19%)	50(57%)	8(9%)	13(15%)	
*+4PQ*	187	55(29%)	103(55%)	13(7%)	16(9%)	0.381

This analysis was done using the Fisher’s exact tests, n = 344

Analysis among *S*. *haematobium* infected and uninfected after excluding those with *S*. *mansoni* and co-infection with both species showed a significant difference in distribution frequency of the *LY* promoter region variant, participants with *LY* promoter genotype being more susceptible to *S*. *haematobium* infection (p = 0.048, Table 6).

**Table 6:** Distribution of participants between *MBL2* genotypes and promoter region haplotypes according to schistosoma infection status.

***MBL2* genotype/ haplotype**	n	***S*. *haematobium* negative**	***S*. *haematobium* positive**	P value
*A/A*	179	51(28%)	128(72%)	
*A/O*	95	33(35%)	62(65%)	
*O/O*	10	4(40%)	6(60%)	0.466
*YAYA/YAXA*	172	49(28%)	123(72%)	
*XAXA/YAYO*	102	35(34%)	67(66%)	
*XAYO/YOYO*	10	4(40%)	6(60%)	0.505
**Promoter genotypes**				
*-550HH*	1	0(0%)	1(100%)	
*-550HL*	33	15(45%)	18(55%)	
*-550LL*	250	73(29%)	177(71%)	0.132
*-550HY*	27	14(52%)	13(48%)	
*-221LY*	250	72(29%)	178(71%)	
*-221LX*	7	2(29%)	5(71%)	0.048
*-221YY*	187	65(35%)	122(65%)	
*-221XY*	89	21(24%)	68(76%)	
*-221XX*	8	2(25%)	6(75%)	0.161
*-550HY*	34	15(44%)	19(56%)	
-221LY*LX	250	73(29%)	177(71%)	0.078
*+4PP*	59	16(27%)	43(73%)	
*+4QQ*	67	17(25%)	50(75%)	
*+4PQ*	158	55(35%)	103(65%)	0.289

This analysis was done using the Chi Square or Fisher’s exact tests, n = 284

Analysis among HIV-1 and *S*. *haematobium* co-infections, after excluding *S*. *mansoni* infections and schistosoma co-infections showed no difference in distribution frequency of MBL genotypes and promoter genotypes between the four co-infection status groups (Table 7). *MBL2* genotypes and promoter region genotypes were not associated with HIV-1 and *S*. *haematobium* co-infections.

**Table 7:** Distribution of participants between *MBL2* promoter region haplotypes according to HIV and *S*. *haematobium* co-infection status.

M ***MBL2* ggenotype/ haplotype**	N	HIV-/***S*. *haematobium***-	HIV+/***S*. *haematobium***+	HIV-/***S*. *haematobium***+	HIV+/***S*. *haematobium***-	P value
*A/A*	179	27(15%)	64(36%)	64(36%)	24(13%)	
*A/O*	95	16(17%)	30(32%)	32(34%)	17(18%)	
*O/O*	10	1(10%)	3(30%)	3(30%)	330(%)	0.802
*YAYA/YAXA*	172	26(15%)	61(36%)	62(36%)	23(13%)	
*XAXA/YAYO*	102	17(17%)	33(32%)	34(33%)	18(18%)	
*XAYO/YOYO*.	10	1(10%)	3(30%)	3(30%)	3(30%)	0.830
**Promoter genotypes**						
*-550HH*	1	0(0%)	0(0%)	1(100%)	0(0%)	
*-550HL*	33	8(24%)	7(21%)	11(33%)	17(21%)	
*-550LL*	250	36(14%)	90(36%)	87(35%)	37(15%)	0.384
*-550HY*	27	8(30%)	5(19%)	8(30%)	6(22%)	
*-221LY*	250	35(14%)	89(36%)	89(36%)	37(15%)	
*-221LX*	7	1(14%)	3(43%)	2(29%)	1(14%)	0.295
*-221YY*	187	32(17%)	59(32%)	64(34%)	32(17%)	
*-221XY*	89	11(12%)	34(38%)	33(37%)	11(12%)	
*-221XX*	8	1(13%)	4(50%)	2(25%)	1(13%)	0.729
*-550HY*	34	8(24%)	7(21%)	12(35%)	7(21%)	
-221LY*LX	250	36(14%)	90(36%)	87(35%)	37(15%)	0.227
*+4PP*	59	7(12%)	20(34%)	23(39%)	9(15%)	
*+4QQ*	67	7(10%)	26(39%)	24(36%)	10(15%)	
*+4PQ*	158	30(19%)	51(32%)	52(33%)	25(16%)	0.685

This analysis was done using the Chi Square or Fisher’s exact tests, n = 284

## Discussion

This study showed that plasma MBL deficiency, all MBL genetic and promoter region variants detected were not associated with HIV-1 infection in this population, however participants with plasma MBL levels at the assay detection limit were significantly more frequent among the HIV-1 infected. Higher plasma MBL levels, *LY* and *LL* promoter genotypes were associated with increased susceptibility to both *S*. *haematobium* and *S*. *mansoni* infections. To our knowledge, this is the first study that has investigated the role of MBL deficiency in HIV-1 and schistosoma co-infections and second study that assessed the role of MBL deficiency in schistosoma infections [59]. Our results also confirm previously reported association of MBL polymorphism with MBL levels [15–17].

In view of available literature, we hypothesized that plasma MBL deficiency due to single locus substitutions in *MBL2* resulting in varying levels of circulating MBL would have an effect on susceptibility to HIV-1 and schistosoma infections in this study population. The results of our study showed no difference in plasma MBL concentration between the HIV positive and HIV negative individuals, consistent with other reports [80–82]. In contrast, some have reported protective effect of normal plasma MBL levels and increased susceptibility due to low MBL levels [30, 37, 83, 84], but some reported a deleterious effect of normal plasma MBL levels where high MBL levels were associated with acquiring HIV infection [39].

In addition, we investigated the role of plasma MBL deficiency in adults singly infected or co-infected with HIV-1, *S*. *haematobium* and *S*. *mansoni* and uninfected controls. Our results showed a significant difference in plasma MBL levels with those *S*. *mansoni* and *S*. *haematobium* positive having significantly higher MBL levels than the uninfected participants. When analysis was done among the *S*. *haematobium* infected and uninfected, after excluding those infected with *S*. *mansoni* and those co-infected with both schistosoma species, the *S*. *haematobium* infected participants had significantly higher plasma MBL levels. The clinical relevance of our findings is that higher plasma MBL levels led to increased susceptibility to schistosoma infections and lower levels were protective. Our results are in contrast to the only similar report available [59] which found lower MBL levels in the infected participants and higher MBL levels were protective against *S*. *haematobium* infection in a Nigerian population [59]. The Nigerian study had a sample size of 346 almost similar to our study with 379 participants. The reasons for these contrasting findings on role of plasma levels in schistosomiasis are not clear but may be due to population differences. There have been reports on the possible advantage of evolution of the *MBL2* variants and the high prevalence of *MBL2* variants resulting in reduced plasma MBL concentrations on the African continent [44, 85]. We can only postulate that selection pressure favouring *MBL2* variants and reduced plasma levels that occurred in African populations offers protection against the numerous intracellular infections present in this population at large. Thus in our study population we found a high prevalence of plasma MBL deficiency at 18% and the reduced plasma MBL levels were found to be protective against schistosoma infections.

Our findings of high plasma MBL deficiency are consistent with findings from other African populations [16, 17, 30]. Population surveys have shown that the concentration of MBL in plasma/serum ranges from <20μg/L to 10 000μg/L [42] and plasma/serum MBL concentration less than 100μg/L is considered deficient [72, 73]. The plasma MBL level median of 800μg/L and the wide range of 20–7600μg/L for our study population are consistent with studies in African populations [17, 30]. Plasma MBL concentration did not differ between males and females nor by age as reported by others [30].

When analysis was done comparing the proportions of those with plasma MBL concentration at the assay detection limit, a strong association with being HIV positive was detected, supporting our hypothesis of MBL consumption and reduction during HIV infection, consistent with other findings [82]. The possible explanation for the association between this severe MBL deficiency and being HIV positive in our study could be that the low MBL levels may indicate that there is consumption of the MBL protein molecule when it engages in opsonic clearance of the HIV virus, leading to MBL consumption [82] and not accumulation as proposed by others [39, 86]. Available evidence shows that MBL assists in HIV clearance through activation of the compliment system and MBL bound to HIV can be cleared from the circulation by the CIq receptor, a molecule that has been shown to have high affinity for MBL [87, 88] and MBL binds to and neutralises HIV in vitro [89]. However, other recent studies have conflictingly shown that MBL levels remain relatively stable during the course of HIV infection and does not support the theory of MBL consumption during HIV infection [82]. We also no differences in *MBL2* levels between CDC HIV categories, CD4^+^ T cell count and viral load in our study, similar to findings by others [39], in contrast to other findings [39].

Plasma MBL concentration was categorised into normal (above 500μg/L), intermediate (100μg/L- 500μg/L) and deficient (below 100μg/L) [72, 73]. Our results showed no association between this categorisation of plasma MBL level, HIV-1 and schistosoma infections. Our results are in contrast to a report that showed increased susceptibility to HIV infection in people with MBL deficiency [30]. We could not find any similar studies comparing these MBL categories with schistosoma infections.

Assessment of *MBL2* polymorphism showed a high prevalence *MBL2* deficient genotypes *A/O* and *O/O* at 18%, due to high frequency of the *C* (G>A) variant allele (20%) and high frequencies of variant promoter alleles X and L. All the currently known *MBL2* alleles, wild-type *A*, *B* (A>G), *C*(A>G), and *D* (C>T) and promoter region alleles *-550H*, *-221Y*, *-550L*, *-221X*, *+4P and +4Q*, were found in this population. Presence of a high frequency of the variant C *MBL2* allele in our population is consistent with findings from other studies on Africans with frequencies as high as 24% in Mozambicans [17], 27% in Gambian adults [45] and 15–38% in several East and West African countries [44, 45]. The MBL genotypes *A/A*, *A/O* and *O/O* in our study correlated with high, intermediate and deficient plasma MBL levels confirming results on other African populations [17, 30]. Promoter region variants have been reported to significantly affect MBL plasma/serum concentration [16], also confirmed by our results. The *HY*, *LY* and *LX* promoters are associated with high, medium and low MBL expression respectively [17]. In our study, the *L* allele (94%) had the highest frequency and *H* allele (5%) had the lowest frequency, consistent with other reports on African populations [16].

We found no association between presence of *MBL2* structural variants and promoter variants with HIV infection, in accordance with other findings [38, 90], in contrast to reports of significant associations [37, 83, 84, 91]. Similar results to ours of no association between *MBL2* polymorphism and HIV infection, reported in a Colombian population, were explained as being due to existence of redundant constitutive acquired immune defence systems that can complement or take up the innate defence functions provided by MBL, which are likely favoured under conditions of high pathogen exposure [38]. It is indeed true that polyparasitism is common in most rural populations in Zimbabwe [61].

There were no differences in distribution of *MBL2* promoter region alleles and variants between the HIV positive and HIV infected participants and no role of MBL promoter region variants in increasing susceptibility to HIV infection, in accord with other reports [92, 93]. In contrast promoter region variants *LX/LX* have been reported to have higher frequency among HIV-infected adults than controls [94]. The *H/Y*, *L/Y* and /*LX* promoters are associated with high, medium and low *MBL2* expression respectively [16]. All the currently known *MBL2* promoter region variants *H*, *L*, *Y*, *X*, *P* and *Q* were detected in our study, the *L* allele (47%) had the highest frequency and *H* allele (3%) had the lowest frequency, in accord with a report on African populations [16]. The *H* allele is found predominantly in white populations who also have very low *L* and *X* alleles [16]. Our results of no association between presence of *MBL2* promoter region variants and HIV infection are in accord with other reports in adults [93] but in contrast and children with promoter type *L* were protected from HIV rapid progression [95].

The reason for the differences in plasma MBL levels, *MBL2* genotypes and HIV association outcomes is not clear [54, 55, 96] but this maybe because studies have been carried out in different populations, homosexual groups [37] and some in heterosexuals [30, 38, 91] and also the methods employed in these studies are different with some measuring plasma MBL levels only [30, 39, 81, 82, 97, 98], others assessing structural alleles only [38, 91, 99], some looked at both [37, 80, 83, 84, 90] others in addition looked at *MBL2* promoter region alleles [92, 100]. Our study investigated all three MBL parameters, plasma MBL levels, polymorphism in the *MBL2* exon 1 gene and promoter region variants. Therefore the conflicting reports on association of HIV infection with MBL levels and genetic polymorphism may be due to differences in HIV transmission route, sample size, ethnicity, environmental conditions and study design as postulated by others [38].

*MBL2* genetic variants *A/O* and *O/O* and the other promoter variants were not associated with schistosoma co-infections in our study similar to findings in a Nigerian population [59]. However our findings showed that the promoter genotypes *L/Y* and /*LL* which code for low plasma levels, both showed significant association with *S*. *haematobium* infection, similar to findings in the Nigerian. In contrast to our findings of no association the promoter genotypes *H/L* and *P/P* were reported to be protective and *P/Q* carriers showed increased susceptibility [59].

Participants were further stratified into six *MBL2* haplotype groups which were combined into three groups for analysis namely, haplotypes that give normal plasma MBL levels (*YA/YA*, *YA/XA*), intermediate levels (*XA/XA*, *YA/YO*) and deficient levels (*XA/YO*, *YO/YO*) [20, 77]. None of the three *MBL2* haplotype groups were associated with HIV-1 nor schistosoma infections in contrast to a report of increased susceptibility where the *XA/XA* promoter variant had a detrimental effect in HIV vertical transmission [100]. Our results showed no association between HIV-1 and schistosoma infections and the seven *MBL2* haplotypes detected in this study, in contrast to findings of significant association between MBL2*HYPA and *S*. *haematobium* infection, those with MBL2*HYPA haplotype were reported to be at a lower risk for *S*. *haematobium* infection, presence of MBL2*HYPA was protective [59].

MBL deficiency has been reported in several studies to be strongly associated with increased susceptibility to several other infections like respiratory infections and recurrent infections in adults and children, infections that are highly prevalent in Zimbabwe. Available literature therefore shows evidence of low MBL levels to be therefore protective in some diseases but increases susceptibility in some infections. This varying evidence shows that the clinical significance of the *MBL2* variants may possibly depend on the population investigated and the type of disease. We found plasma MBL deficiency and *MBL2* gene and promoter region variants to play no role in HIV-1 infection, but high plasma MBL levels and the heterozygous promoter genotype *LY* and homozygous *LL* increased susceptibility to *schistosoma* infections.

The main limitation of our study was inclusion of few men in the main MUSH study. There is a gold panning area in the neighbouring district, most men work away from their rural homes and were not available during our study recruitment phase. This has the potential to bias our findings but every effort was done to enrol all eligible men into the main MUSH study and MBL sub-study.

In conclusion, this study showed a high prevalence of plasma MBL deficiency, high frequency of *MBL2* genetic variant *C* (G>A). These findings are consistent with other observations that the *C* (G>A) variant allele is the predominant variant allele in Sub-Saharan Africa. We found no evidence of an association between MBL deficiency and HIV-1 infection, however plasma MBL levels at assay detection limit were associated with HIV-1 infection indicating a possible role of MBL deficiency in HIV infection. Lower plasma MBL levels were protective against both *S*. *haematobium* and *S*. *mansoni* infections and higher plasma MBL levels associated with increased susceptibility to schistosoma infections. All the other MBL gene and promoter region variants detected played no role in both HIV and schistosoma infections but presence of promoter region variants *LY* and *LL* increased susceptibility to both *S*. *haematobium* and *S*. *mansoni* infections. The available evidence on the association of polymorphism in the *MBL2* promoter region with HIV-1 infection is still conflicting. In light of all this conflicting evidence it would be difficult to recommend the use of MBL plasma levels, *MBL2* structural variants and promoter region mutations as a biomarker of HIV infection and for the monitoring of ARV therapy together with viral load and CD4^+^ T cell lymphocyte counts in the population represented by our study participants. Our results on role of plasma MBL levels in schistosoma infections are in conflict with the only available report that found lower plasma MBL levels to increase susceptibility to schistosoma infections. There is therefore, need to carry out a much bigger study to verify these results. In addition, future immunological studies looking at the association between MBL deficiency and other diseases are highly recommended for the Zimbabwean population where persistent recurrent bacterial, viral, fungal and other parasitic infections remain prevalent affecting both adults and children.

## Supporting Information

S1 Dataset MS Excel format.xls. Data set showing study variables HIV-1, schistososma, plasma MBL and *MBL2* genotyping results.
(XLSX)

S1 Fig Box Plot showing differences in median plasma MBL levels according to *MBL2* genotype. *MBL2* genotypes were categorized into three functional groups A/A, A/O and O/O. There was a statistically significant difference in median plasma MBL concentration between the 3 genotype groups, with the A/A genotype having the highest median plasma MBL concentration and O/O the lowest (AA: 1552μg/L, A/O: 139μg/L, O/O: 20μg/L, p<0.0001). Differences in median MBL concentration by *MBL2* genotype were analysed by Kruskal-Wallis non-parametric tests (n = 366). IQR = Interquartile range.
(TIFF)

S2 Fig Box Plot showing comparison of median plasma MBL levels according the seven main *MBL2* haplotypes found in this study. HYPA haplotype showed the highest median plasma MBL concentration (median MBL 2464μg/L, IQR 1336–3368μg/L) and LYQC haplotype had the lowest levels, at assay detection limit of 20μg/L. We found varying median MBL concentrations due to the effect of HY, LY and LX promoters.
(TIFF)

S3 Fig Box Plot showing *MBL2* genotypes and plasma MBL concentration. The *MBL2* genotypes were further combined and subdivided into three groups namely genotypes that give normal plasma MBL levels (YA/YA, YA/XA), intermediate levels (XA/XA, YA/YO) and deficient levels (XA/YO, YO/YO) (20, 77). There was a statistically significant difference in median plasma MBL concentration between the 3 haplotype groups, with the (YA/YA, YA/XA) haplotypes showing the highest median plasma MBL concentration1568μg/L, followed by (XA/XA, YA/YO) (151μg/L) and (XA/YO, YO/YO) (20μg/L) showing deficient levels (p<0.0001).
(TIFF)

S1 Table The 12 oligonucleotide primer sequences and the Exon 1 internal control primers, that were used in this study for detection of *MBL2* coding and promoter normal and variant alleles. These primer sequences and specifications were for identification of the *MBL2* and promoter region types, according to manufacturer’s instructions (DNA Technology, Denmark).
(DOCX)

S2 Table *MBL2* genotypes and promoter SNPs and HWE. The HWE for the above *MBL2* SNPs was determined among the HIV negative participants.
(DOCX)

S3 Table Detailed summary of the *MBL2* genotypes, haplotypes and corresponding plasma MBL concentrations (n = 366). Gene and allele frequencies were obtained by direct gene counting. The three main *MBL2* genotype groups AA, AO and OO were further subdivided according to the *MBL2* gene and haplotype combinations detected. Twenty-four different complete *MBL2* genotypes were detected as shown above. As expected, the HYPA/HYPA genotype which codes for the homozygous normal A/A *MBL2* genotype, had the highest median plasma MBL concentration (median MBL 2464μg/L, IQR 1336–3368μg/L) and LYQC/LYQC had the lowest levels, (MBL 20μg/L). HYPA haplotype showed the highest median plasma MBL concentration (median MBL 2464μg/L, IQR 1336–3368μg/L) and LYQC haplotype had the lowest levels (MBL 20μg/L). We found varying median MBL concentrations due to the effect of HY, LY and LX promoters.
(DOCX)
