# Supplementary material for: Role of Mannose-Binding Lectin Deficiency in HIV-1 and Schistosoma Infections in a Rural Adult Population in Zimbabwe
Source: PLoS One. 2015 Apr 1;10(4):e0122659. doi: 10.1371/journal.pone.0122659 (PMC4382150; doi:10.1371/journal.pone.0122659)
Supplement: S1 Table — These primer sequences and specifications were for identification of the MBL2 and promoter region types, according to manufacturer’s instructions (DNA Technology, Denmark). (DOCX) [file pone.0122659.s005.docx]

##### Table S1. The 12 oligonucleotide primer sequences and the Exon 1 internal control primers, that were used in this study for detection of *MBL2* coding and promoter normal and variant alleles.

|  | **primer** | **Specific sequence** | **Product**  **Size (bp)** |
| --- | --- | --- | --- |
| 1 | A54  none B | 5´-AGTCGACCCAGATTGTAGGACAGAG-3´  3´-CCTTTTCTCCCTTGGTGC-5´ | 278 |
| 2 | B | 5´-GGGCTGGCAAGACAACTATTA-3´  3´-GCAAAGATGGGCGTGATGA-5´ | 278 |
| 3 | A57  None C | 5´-AGTCGACCCAGATTGTAGGACAGAG-3´  3´-CCTGGTTCCCCCTTTTCTC-5´ | 290 |
| 4 | C | 5´-AGTCGACCCAGATTGTAGGACAGAG-3´  3´-ACCTGGTTCCCCCTTTTCTT-5´ | 290 |
| 5 | A52  none D | 5´-AGTCGACCCAGATTGTAGGACAGAG-3´  3´-TCCCTTGGTGCCATCACG-5´ | 268 |
| 6 | D | 5´-AGTCGACCCAGATTGTAGGACAGAG-3´  3´-CTCCCTTGGTGCCATCACA-5´ | 268 |
| 7 | Promoter X | 5´-CTCAGGGAAGGTTAATCTCAG-3´  3´-CATTTGTTCTCACTGCCACC-5´ | 440 |
| 8 | Promoter Y | 5´-CTCAGGGAAGGTTAATCTCAG-3´  3´-CATTTGTTCTCACTGCCACG-5´ | 443 |
| 9 | Promoter H | 5´-GGCTTAGACCTATGGGGCTA-3´  3´-GCTTCCCCTTGGTGTTTTAC-5´ | 316 |
| 10 | Promoter L | 5´-GGCTTAGACCTATGGGGCTA-3´  3´-GCTTCCCCTTGGTGTTTTAG-5´ | 316 |
| 11 | P | 5´-AGGATCCAGGCAGTTTCCTCTGGAAGG-3´  3´-TAGGACAGAGGGCATGCTC-5´ | 335 |
| 12 | Q | 5´-AGGATCCAGGCAGTTTCCTCTGGAAGG-3´  3´-TAGGACAGAGGGCATGCTT-5´ | 331 |
|  | Exon 1 internal  control | 5´-GAGTTTCACCCACTTTTTCACA-3´  3´-GCCTGAGTGATATGACCCTCC-5´ | 431 |

___________________________________________________________________________

These primer sequences and specifications were for identification of the *MBL2* and promoter region types, according to manufacturer’s instructions (DNA Technology, Denmark).
