# Supplementary material for: Role of Mannose-Binding Lectin Deficiency in HIV-1 and Schistosoma Infections in a Rural Adult Population in Zimbabwe
Source: PLoS One. 2015 Apr 1;10(4):e0122659. doi: 10.1371/journal.pone.0122659 (PMC4382150; doi:10.1371/journal.pone.0122659)
Supplement: S2 Table — The HWE for the above MBL2 SNPs was determined among the HIV negative participants. (DOCX) [file pone.0122659.s006.docx]

**Table S2.** *MBL2* genotypes and promoter SNPs and HWE

**________________________________________________________________________**

| ***MBL2* variant** | **Reference SNP ID number (rs#)** | **Alleles** | **Alternative nomenclature** | **Location** | **HWE controls P** |
| --- | --- | --- | --- | --- | --- |
| -550 | 11003125 | G/C | H/L | promoter | 0.830 |
| -221 | 7096206 | C/G | X/Y | promoter | 0.550 |
| +4 | 7095891 | A/G | P/Q | Exon 1 | 0.170 |
| Exon 1  B(codon 54)  C(codon 57)  D(codon 52) | 1800450  1800451  5030737 | A/G  G/A  C/T | A/B  A/C  A/D | Exon 1 | 0.400 |

__________________________________________________________________________-

The HWE for the above *MBL2* SNPs was determined among the HIV negative participants.
