# Supplementary material for: Role of Mannose-Binding Lectin Deficiency in HIV-1 and Schistosoma Infections in a Rural Adult Population in Zimbabwe
Source: PLoS One. 2015 Apr 1;10(4):e0122659. doi: 10.1371/journal.pone.0122659 (PMC4382150; doi:10.1371/journal.pone.0122659)
Supplement: S3 Table — Gene and allele frequencies were obtained by direct gene counting. The three main MBL2 genotype groups AA, AO and OO were further subdivided according to the MBL2 gene and haplotype combinations detected. Twenty-four different complete MBL2 genotypes were detected as shown above. As expected, the HYPA/HYPA genotype which codes for the homozygous normal A/A MBL2 genotype, had the highest median plasma MBL concentration (median MBL 2464μg/L, IQR 1336–3368μg/L) and LYQC/LYQC had the lowest levels, (MBL 20μg/L). HYPA haplotype showed the highest median plasma MBL concentration (median MBL 2464μg/L, IQR 1336–3368μg/L) and LYQC haplotype had the lowest levels (MBL 20μg/L). We found varying median MBL concentrations due to the effect of HY, LY and LX promoters. (DOCX) [file pone.0122659.s007.docx]

**Table S3.** Detailed summary of the *MBL2* genotypes, haplotypes and corresponding plasma MBL concentrations (n=366)

___________________________________________________________________________

| **Structural genotype** | **Promoter** | **Complete genotype** | **(n)** | **MBL concentration median µg/L** | **MBL concentration IQR (µg/L)** |
| --- | --- | --- | --- | --- | --- |
| AA | HY/HY | HYPA/HYPA | 4 | 2464 | 1336-3368 |
|  | LYLY | LYPA/LYPA | 16 | 1149 | 672-2280 |
|  |  | LYQA/LYQA | 47 | 2048 | 1344-2960 |
|  |  | LYPA/LYQA | 59 | 1856 | 992-3024 |
|  | LX/LX | LXPA/LXPA | 12 | 856 | 568-1760 |
|  | HY/LY | HYPA/LYPA | 3 | 656 | 136-2080 |
|  |  | HYPA/LYQA | 13 | 1520 | 1088-1888 |
|  |  | HYPA/LXPA | 5 | 1680 | 720-2400 |
|  | LY/LX | LYPA/LXPA | 27 | 1040 | 688-2000 |
|  |  | LYPA/LXQA | 42 | 1016 | 752-1904 |
|  |  | LYQA/LXQA | 1 | - | 286 |
| A/O | HY | HYPD/LYPA | 1 | - | 1024 |
|  |  | HYPA/LYQC | 10 | 244 | 20-352 |
|  |  | HYPA/LXQC | 1 | - | 192 |
|  | LY | LYPA//LYPB | 1 | - | 512 |
|  |  | LYPA/LYQB | 1 | - | 225 |
|  |  | LYPA/LYQC | 48 | 150 | 24-416 |
|  |  | LYQA/LYQB | 1 | - | 202 |
|  |  | LYQA/LYQC | 26 | 188 | 105-528 |
|  | LX | LYPA/LXPC | 2 | 162 | 62-263 |
|  |  | LYPA/LXQC | 27 | 27 | 20-83 |
|  |  | LYQA/LXQC | 3 | 128 | 20-1920 |
| O/O |  | LYQC/LYQC | 12 | 20 | 20-20 |
| **Haplotypes** |  |  |  |  |  |
| HYPA | HY |  | 36 | 1024 | 310-1992 |
| HYPD | HY |  | 1 | - | 1024 |
| LYQA | LY |  | 85 | 1056 | 346-2320 |
| LYPA | LY |  | 217 | 816 | 163-1936 |
| LXPA | LX |  | 12 | 856 | 568-1760 |
| LYPB | LY |  | 2 | 368 | 225-512 |

______________________________________________________________________________________________________________

Gene and allele frequencies were obtained by direct gene counting. The three main *MBL2* genotype groups AA, AO and OO were further subdivided according to the *MBL2* gene and haplotype combinations detected. Twenty-four different complete *MBL2* genotypes were detected as shown above. As expected, the HYPA/HYPA genotype which codes for the homozygous normal A/A *MBL2* genotype, had the highest median plasma MBL concentration (median MBL 2464µg/L, IQR 1336-3368µg/L ) and LYQC/LYQC had the lowest levels, (MB2 20µg/L). HYPA haplotype showed the highest median plasma MBL concentration (median MBL 2464µg/L, IQR 1336-3368µg/L ) and LYQC haplotype had the lowest levels (MBL 20µg/L). We found varying median MBL concentrations due to the effect of HY, LY and LX promoters.
